# Forest litter crickets prefer higher substrate moisture for oviposition: Evidence from field and lab experiments

**DOI:** 10.1371/journal.pone.0185800

**Published:** 2017-10-04

**Authors:** Fernando de Farias-Martins, Carlos Frankl Sperber, Daniel Albeny-Simões, Jennifer Ann Breaux, Marcos Fianco, Neucir Szinwelski

**Affiliations:** 1 Laboratório de Orthoptera, Universidade Estadual do Oeste do Paraná, Cascavel, Paraná, Brazil; 2 Programa de Pós-Graduação em Conservação e Manejo de Recursos Naturais, Universidade Estadual do Oeste do Paraná, Cascavel, Paraná, Brazil; 3 Departamento de Biologia Geral, Universidade Federal de Viçosa, Viçosa, Minas Gerais, Brazil; 4 Programa de Pós-Graduação em Ciências Ambientais, Universidade Comunitária da Região de Chapecó, Chapecó, Santa Catarina, Brazil; 5 Instituto Latino-Americano de Ciências da Vida e da Natureza, Universidade Federal da Integração Latino-Americana, Foz do Iguaçu, Paraná, Brazil; Museum National d’Histoire Naturelle, FRANCE

## Abstract

For insects, choosing a favorable oviposition site is a type of parental care, as far as it increases the fitness of its offspring. Niche theory predicts that crickets should show a bell-shaped oviposition response to substrate moisture. However, lab experiments with mole crickets showed a linear oviposition response to substrate moisture. Studies with the house cricket *Acheta domesticus* also showed a linear juvenile body growth response to water availability, thus adult ovipositing females should respond positively to substrate moisture. We used a field experiment to evaluate the relationship between oviposition preference and substrate moisture in forest litter-dwelling cricket species. We also evaluated oviposition responses to substrate moisture level in *Ubiquepuella telytokous*, the most abundant litter cricket species in our study area, using a laboratory study. We offered cotton substrate for oviposition which varied in substrate moisture level from zero (i.e., dry) to maximum water absorption capacity. We used two complementary metrics to evaluate oviposition preference: (i) presence or absence of eggs in each sampling unit as binary response variable, and (ii) number of eggs oviposited per sampling unit as count response variable. To test for non-linear responses, we adjusted generalized additive models (GAMM) with mixed effects. We found that both cricket oviposition probability and effort (i.e., number of eggs laid) increased linearly with substrate moisture in the field experiment, and for *U. telytokous* in the lab experiment. We discarded any non-linear responses. Our results demonstrate the importance of substrate moisture as an ecological niche dimension for litter crickets. This work bolsters knowledge of litter cricket life history association with moisture, and suggests that litter crickets may be particularly threatened by changes in climate that favor habitat drying.

## Introduction

The distribution of organisms in an environment is influenced simultaneously by top-down and bottom-up control mechanisms [1, 2], synthesized in the concepts of the ecological niche. Ecological niche theory predicts that competing organisms have an optimum range of abiotic conditions, outside of which, results for fitness can be sub-optimal, hazardous or even lethal at extreme values [3].

The impacts of bottom-up control on population dynamics in oviparous insects are strongly influenced by maternal oviposition site choice, which can impact both maternal and offspring survival [4–6]. Maternal survival can be enhanced by avoiding exposure time to potential predators during breeding or egg laying events, by maximizing the number of eggs laid rather than egg quality, and by avoiding harsh environments [4, 7]. To maximize offspring survival, females should prefer to oviposit in sites where egg predation risk [8], desiccation, and freezing [9, 10] is low, sites with temperatures within the optimal range for egg hatching of that species [8], and sites with adequate resource availability for the developing eggs and emerged juveniles [11]. These habitat features positively influence eclosion rates [12] and offspring survival probability [5, 13] and consequently, net reproductive rates [14].

Substrate moisture is among the most important bottom-up factors to oviposition site selection [15, 16], because it exerts effects on physiology, development, and metabolism [17, 18]. Water limitation, especially during embryonic and juvenile stages, can hinder chitin synthesis and ecdysis in arthropods [19], reduce body size and mass [20–22], alter pigmentation [23], and hinder locomotion [24, 25], and may affect species distributions and abundances [18]. Excess of water can be lethal, due to pathogen development [26, 27], freezing [28] or drowning [27]. However, few studies have investigated effects of substrate moisture on insect oviposition preference [13, 15, 16, 29], instead focusing on effects of temperature on reproductive patterns [5, 8, 30, 31]. For litter crickets (Orthoptera: Grylloidea), factors known to affect oviposition preference include chemical compounds in male sperm [32], neural patterns regulated by ovipositor sensilla [33], and temperature [34]. However, to our knowledge, litter cricket oviposition site choice and egg laying frequency in response to substrate moisture has not yet been assessed. Once oviposition behavior is a crucial element of insect fitness, it is important to understand where crickets choose to place their eggs.

Linking environment and genotype, phenotypic plasticity [35] and reaction norms [36, 37] are proximal explanations for oviposition preference in response to environmental conditions, such as substrate moisture. Phenotypic plasticity is defined as the capacity of a genotype to produce different phenotypes as a result of environmental interactions [38], while reaction norms describe the distribution of those phenotypes across varying environments [39]. In either case, (ecological niche or plasticity/reaction norms), unimodal distribution of phenotypes is predicted across a gradient of environmental moisture, with lower oviposition rates in environmental extremes. We would then expect a non-linear, bell-shaped oviposition response to a gradient of water absorption capacity substrate ranging from zero to 100%. However, a laboratory study with mole crickets indicated a linear oviposition responses to substrate moisture [29], and a study with house crickets (*Acheta domesticus*) indicated a linear growth response to water availability [20], suggesting the possibility of a linear increase in oviposition in response to substrate moisture.

Here we evaluated, through manipulative experiments in field and lab, the preference of cricket oviposition in relation to substrate moisture, testing two alternative hypotheses: (i) oviposition preference shows a non-linear response to substrate moisture, following classical niche theory/norm of reaction predictions; or (ii) oviposition increases linearly with substrate moisture, following available evidence found for other cricket species.

## Materials and methods

### Study organisms

Crickets present high diversity in neotropical forests, where they occur from ground level to the tree canopy, being particularly abundant in forest litter [40]. Crickets are oviparous, hemimetabolous insects that oviposit in soil, litter and plant tissues [41]. Litter crickets are recognized as omnivores, with a primarily herbivorous diet, supplemented with animal tissue, fungi and fruits [41]. Juvenile instars generally share the same habitat and resources as adults [42]. Most crickets hide during the day under fallen logs, rocks, leaf litter or in holes in the ground. Singing species stridulate loudly on warm nights, especially after the rain [43]. Litter crickets have a narrow tolerance range in terms of humidity [20], available resources [44, 45], specific habitat requirements, and spatial heterogeneity [46]. This dependency on multiple environmental factors may result in a strong response to forest regeneration [47].

The lab experiment was done with adult females of *Ubiquepuella telytokous* Fernandes, 2015. This species is the most abundant litter cricket in the study area [45, 47] throughout the year. Very little is known about *U. telytokous* biology, except its habit to walk very quickly on the lower part of tree trunks.

Authorization for collection in the Iguaçu National Park was granted by Instituto Chico Mendes de Conservação e Biodiversidade—ICMBio for NS (SISBIO 46964). These cricket groups are not red listed as threatened nor under risk of extinction.

### Field experiment

**Fig 1:**
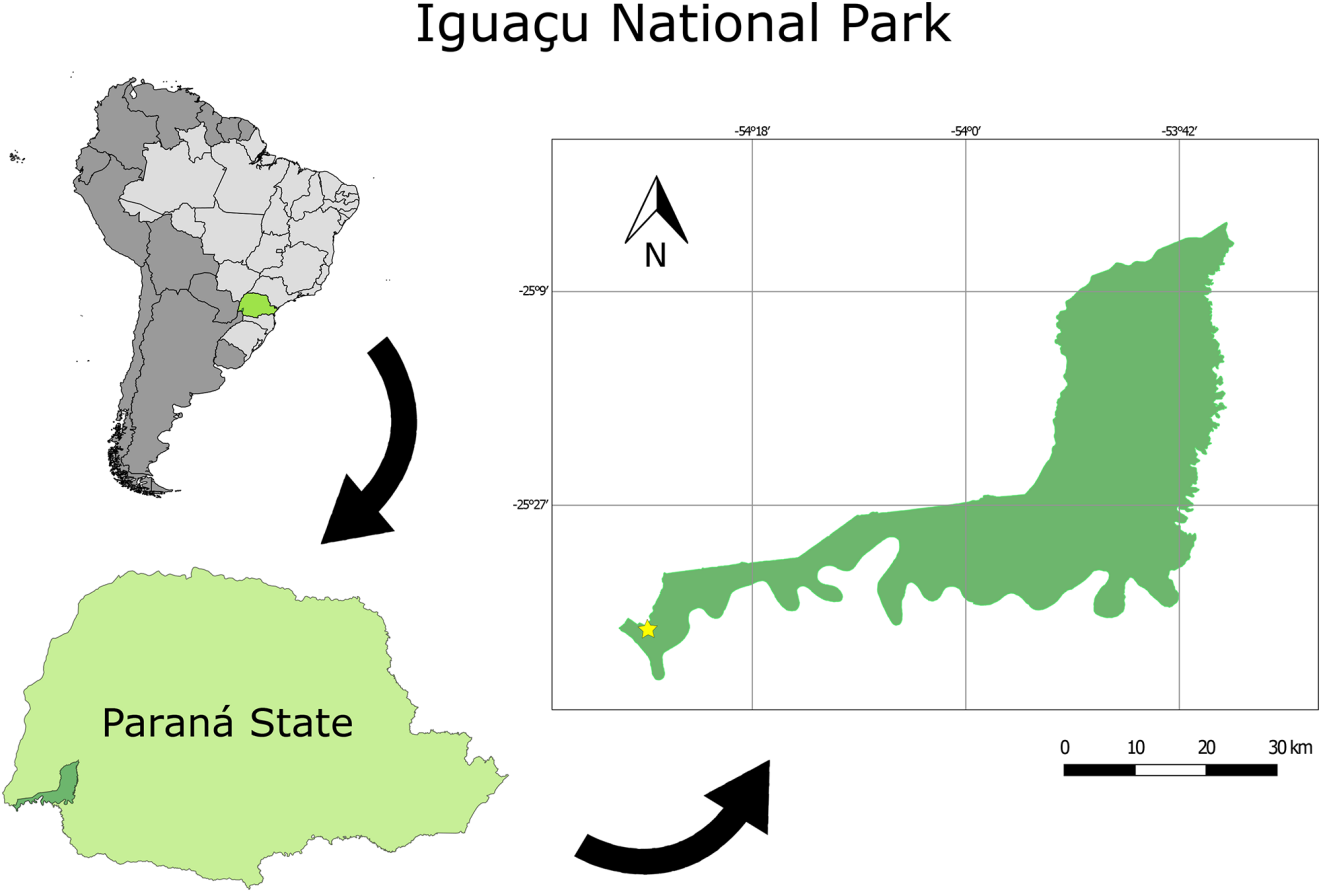
Iguaçu National Park, Foz do Iguaçu, PR, Brazil. The star represents the geographic coordinates of the experiment location.

The field experiment was carried out in May 2012 in an old-growth Atlantic forest at the Iguaçu National Park (25°37’35”S—54°27’9”W) in Foz do Iguaçu, Paraná, Brazil (Fig 1). The vegetation of Iguaçu National Park is composed of tropical semi-deciduous forest and ombrophilous mixed forest, and lies within the Atlantic rain-forest biome. The regional climate is categorized as humid subtropical mesothermal, with a mean annual temperature of 19°C and mean annual rainfall around 1600 mm [48].

**Fig 2:**
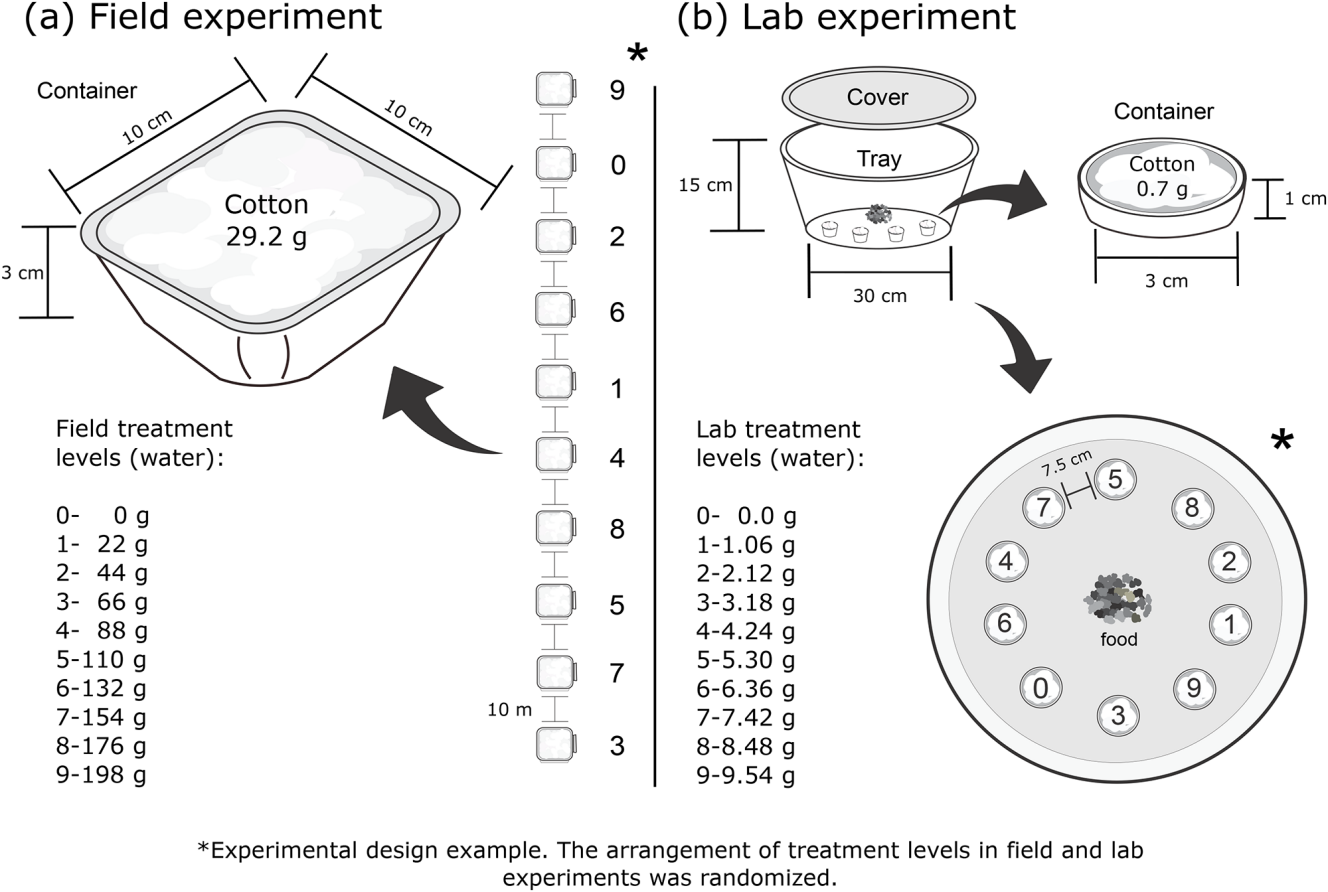
Experimental design for field (a) and lab (b) experiments. Arrangement of moisture levels in both experiments was randomized.

Thirty parallel transects were established inside the forest, placed 500 m from the edge. Each transect was 90 m in length, and the distance between transects was 30 m. Ten plastic containers (10 x 10 x 3 cm) were placed 10 m apart along each transect (total sampling effort = 300 containers; true replicate number = 30). Each transect included ten treatment levels for substrate moisture, ranging from 0% moisture (i.e., dry) to 100% water absorption capacity of the cotton substrate. This was achieved by filling each container with the maximum capacity (29.2 g) of commercial hydrophilic cotton (Algodão Nathalya, Abreu e Lima, PE, Brazil), and pouring from zero to 198 g of water on the cotton substrate, effectively increasing water weight by 22 g per treatment level (see Fig 2a). The highest volume water addition corresponded to 100% absorption capacity of the substrate.

Each container was buried in the ground with the opening at litter level. The order of placement for moisture level treatments along transects was randomized. Containers remained in the field for 48 h to allow litter cricket oviposition. Containers were then collected, packaged, and transported to the lab, and the cotton substrate from each container was weighed to estimate water evaporation in the field. Grylloidea eggs on cotton substrates were then identified and counted using a stereo microscope. Eggs were identified based on specific morphological characteristics, including having pale-yellow coloration and fusiform shape with rounded edges [49, 50].

### Laboratory experiment

The laboratory oviposition experiment took place in a climate-controlled room at 25°C with 80% relative moisture and a 12:12 L:D photoperiod. Experiments were conducted using adults of the parthenogenetic cricket *U. telytokous*, collected from Iguaçu National Park, where they are highly abundant [45, 47]. *U. telytokous* were kept in a 150 x 50 x 50 cm terrarium with abundant food and water. The terrarium was kept in a climate-controlled room for acclimation two days prior to the beginning of oviposition trials.

The lab experiment was essentially a replication of the field experiment on a smaller scale, with controlled environmental conditions and using *U. telytokous* as the model organism. We established multiple-choice arenas using transparent, circular trays (30 cm radius x 15 cm height), which corresponded to the transects in the field experiment (Fig 2b). We used trays that were circular in shape to avoid effects of cricket preferences for corners (N. Szinwelski, pers. obs.). Ten small containers (3 cm diameter x 1 cm height) were arranged radially inside of each arena (Fig 2b), each filled with 0.7 g of commercial hydrophilic cotton. The treatments consisted of ten moisture levels, ranging from 0% to 100% water absorption capacity of the cotton substrate, which corresponded to an increase of 1.06 g water weight per treatment level (max: 9.54 g of water added). As in the field experiment, the order of moisture level treatments within the arena (transect) was randomly chosen.

In the center of each circular tray, we placed three grams of fish food flakes [41]. After assembling the multiple-choice arenas, we chose the thirty adult females with the highest body masses, as heavier individuals tend to be more fertile [51] and are more likely to oviposit in a laboratory setting. Trials were carried out using a single female to avoid competition and potential harm by competing females. Females were placed in the center of trays, which were covered tightly with plastic covering to prevent escape and minimize water evaporation.

After 48 h female crickets were sacrificed, fixed in ethanol [52] and deposited in the Orthoptera Laboratory of the Museu Regional de Entomologia at the Universidade Federal de Viçosa. We weighed the cotton substrate of each container to estimate water evaporation over the experimental period (48 h), and counted eggs.

### Data analysis

We used two complementary metrics to evaluate oviposition preference: (i) presence or absence of eggs in each sampling unit as a binary response variable, and (ii) number of eggs oviposited per sampling unit as a count response variable. The binary approach provides information about female cricket preferences for moisture level for oviposition, while count data indicate effort expended (i.e., the number of eggs laid). Mixed effects models were fit with random intercept [53] in all statistical models, and true replicates (a transect with 10 containers for the field experiment; circular arena for the lab experiment) were treated as random effects. This random effect was used to account for spatial autocorrelation of nearby containers within the same transect in the field experiment, as well as for behavioral autocorrelation of the same individual on each multiple-choice arena in the lab experiment. Although spatial autocorrelation in the field is less likely than behavioral autocorrelation in the lab, we used the same statistical approach for sake of symmetry.

To test whether oviposition preference followed a non-linear, bell-shaped response to substrate moisture, we adjusted generalized additive mixed models (GAMMs) [54] with substrate moisture as an integer smooth term varying from zero (no water added) to nine (100% water absorption capacity of the cotton substrate). We adjusted alternative GAMMs varying the k value (knots) from two to 10 to account for eventual bias in the adjusted results [54]. The GAMMs were adjusted separately for each experiment and response variable (binomial and number of oviposited eggs). If there was evidence of non-linearity, the fitted GAMM should generate an estimate for degrees of freedom (e.d.f.) that is significantly higher than one [54]. If non-linearity was excluded, then we adjusted generalized linear mixed models (GLMMs) with the following explanatory terms: moisture (integer value from zero to nine, for the sake of symmetry between field and lab experiment), experiment site (two levels: field and lab), the experiment x moisture interaction, and evaporation (= initial—final weight (g) of the substrate). Again, random effect was the true replicate.

To analyze presence or absence of oviposition as response variable, we fitted binomial GLMMs with the canonical logit link function and binomial residual distribution [55]. To analyze number or oviposited eggs as response variable we fitted Poisson GLMMs with the canonical log link function and Poisson residual distribution. If overdispersion was detected, we fitted negative binomial GLMMs. The adjusted models were subjected to residual analysis to evaluate model suitability. All statistical analyses were performed in R version 3.4.0 [56]. Raw data are provided in the supporting information (S1 Table).

## Results

The field oviposition experiment yielded 229 Grylloidea eggs in total, while the lab experiment yielded a total of 41 (Table 1). The number of eggs per treatment (substrate) varied from zero to four (field) or zero to seven (laboratory). In the field experiment, 179 substrate units (60%) had no eggs, 51 units had one single egg, 40 units had two eggs, 22 units had three eggs, and eight units had four eggs. In the lab experiment, only eight females (30%) oviposited in more than one substrate unit; there were 267 units (89%) with no eggs, 30 units with a single egg, two units with two eggs, and one substrate unit with seven eggs.

**Table 1:** Numbers of eggs oviposited on cotton substrate (values summed per moisture level) after 48 hours in field and lab experiments.

Moisture levels	Egg number	
	Field	Lab
0	3	0
1	3	1
2	9	0
3	6	0
4	12	5
5	29	1
6	44	1
7	44	5
8	39	17
9	40	11
**Total**	**229**	**41**

Overall, fewer eggs were deposited in lower moisture substrates (Table 1). In the field experiment, the three highest moisture levels accumulated 123 eggs (54%). The next three highest moisture levels three accumulated 85 eggs (37%), and the four substrates with lowest moisture levels accumulated 21 eggs (9%). In the lab experiment, the three highest moisture levels accumulated 33 eggs (80%), while the next highest three moisture levels accumulated seven eggs (17%); the four substrates with lowest moisture levels contained only a single egg (2%), which was deposited on the substrate treatment with the second-lowest moisture level.

**Fig 3:**
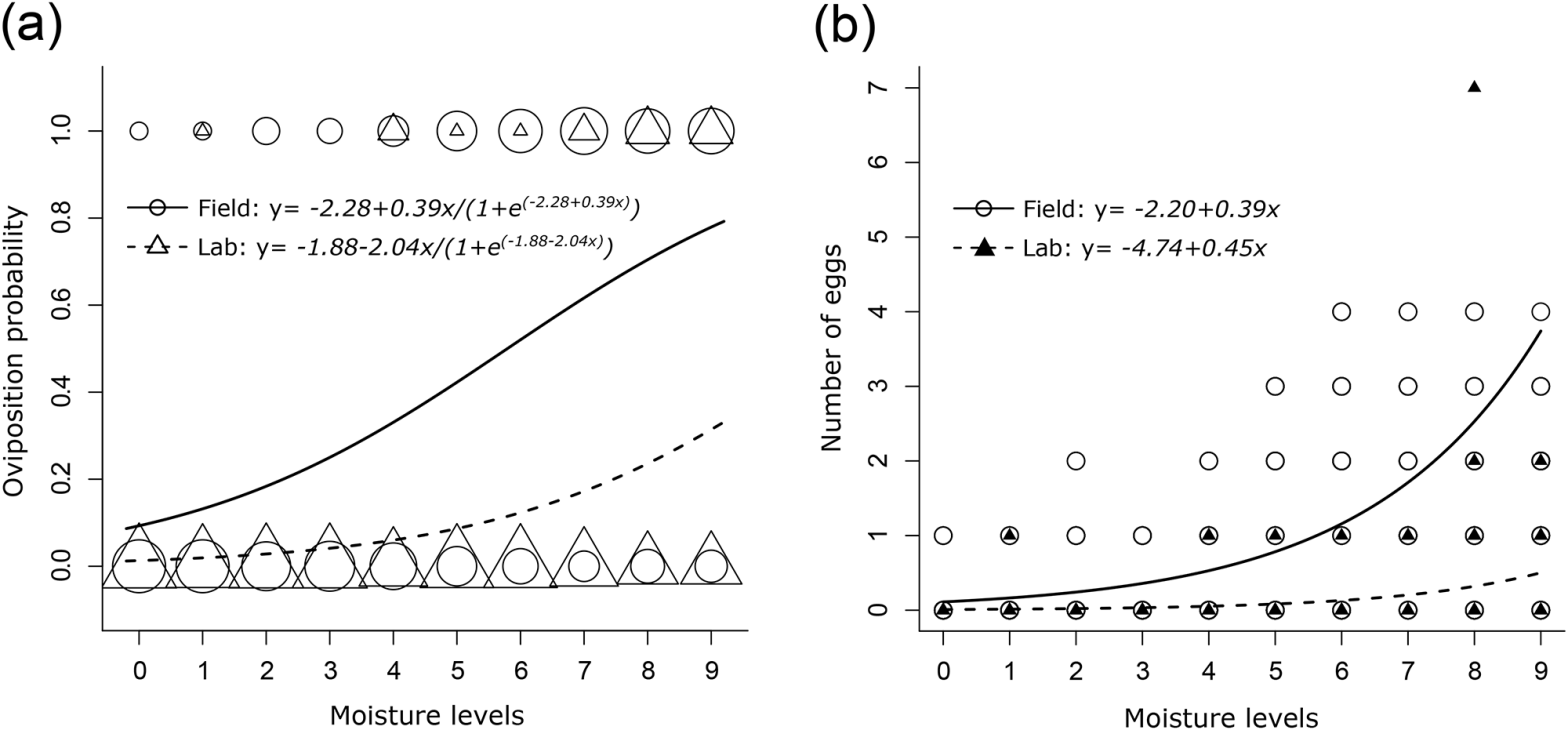
Cricket oviposition responses to substrate moisture level. (a) represents oviposition probability, a binary response variable with a value of either 0 (if oviposition did not occur in any replicate containers for that moisture level) or 1 (if oviposition occurred at least one of the container replicates in that level). Circles represent the field experiment, while triangles represent the lab experiment. The size of circles and triangles represents the number of observations (= the number of replicates in which the binary response occurred) for the same moisture level. Curves represent the minimal adequate model of the adjusted logistic regression (solid line for field experiment, dashed line for lab experiment; n = 30 for each experiment). (b) Numbers of eggs deposited per container by moisture level in field (circles) and lab (filled triangles) experiments. Curves represent the minimal adequate model of the adjusted regression (GLMM with negative binomial distribution) for field (solid line) and lab (dashed line) experiments (n = 30 for each experiment). The equations represent the estimated parameters for each model.

In the field experiment, 90.83% of the eggs were oviposited on substrate with moisture higher than 44%; in the lab experiment that figure rose to 97.56%. We found no evidence for non-linear effects of moisture on oviposition probability, nor on number of oviposited eggs (e.d.f. varied between 0.999 and 1.001) [57] for field or lab data (P > 0.05). No overdispersion was detected in the binomial models. There were no significant interactions between experiment site (field or lab) and moisture level, or for site and oviposition probability (*χ*^2^ = 0.43, P = 0.5) or number of eggs oviposited (*χ*^2^ = 1.55, P = 0.2). Evaporation in the field experiment over the 48 h oviposition period ranged from 0 to 16%. There was no evaporation in the lab. Evaporation reduced the number of oviposited eggs in the field (*χ*^2^ = 104.91, P < 0.001). The probability of oviposition was significantly higher in the field experiment than in the lab (*χ*^2^ = 51.20, P < 0.001, Fig 3a).

For both experiments, the probability of oviposition increased linearly with moisture level (*χ*^2^ = 83.76, P < 0.001, Fig 3a). Numbers of eggs were significantly lower in the laboratory experiments (*χ*^2^ = 99.38, P < 0.001, Fig 3b). Egg numbers increased linearly with moisture level in the field experiment (*χ*^2^ = 77.65, P < 0.001, Fig 3b). Evaporation reduced the number of eggs oviposited in the field (*χ*^2^ = 104.91, P < 0.001), however, evaporation did not alter the probability of oviposition (*χ*^2^ = 1.55, P = 0.2). The preference for moist substrates was more accentuated in the lab than in the field, as depicted by the higher estimates for the slopes for both oviposition probability and number of oviposited eggs (Fig 3).

## Discussion

Although we did not identify eggs to species level in the field experiment, we positively identified all eggs as belonging to Grylloidea, and data from other published studies in the same site have demonstrated that *U. telytokous* is by far the most abundant species in the area (cited as *Ectecous* sp1 in the original paper [47]). In this study, 650 *U. telytokous* were captured (55% of all sampled crickets in that study), while for the second and third most abundant species (*Phoremia* sp1 and *Lerneca* sp1), only 105 (9%) and 104 (9%) individuals were captured, respectively [47]. In another study, 563 collected individuals (50% of all sampled crickets in that study) were *U. telytokous* (again cited as *Ectecous* sp1 in the original paper [45]), in contrast to 215 *Phoremia zefai* Pereira, Sperber & Lhano, 2011 (19%) and 130 *Aracamby* sp. (12%). Hence, although the eggs oviposited in the field were not identified to species level, this does not weaken our conclusions on forest litter cricket oviposition preferences in general.

Litter cricket oviposition was positively and linearly correlated to substrate moisture, indicating that moisture is an important niche dimension for these organisms. The observed linear response showed that, within the range of moisture levels of this study, there are no negative effects of extreme high values of substrate moisture. However, if our moisture range included standing water, i.e., water exceeding substrate capacity, it might have shown an unimodal response. In other words, the highest levels of moisture in this experiment may have been recognized as only intermediate moisture by the animals. In the field, it is common to find flooded regions, were water level exceeds substrate capacity. In such sites, we expect that oviposition is precluded.

Substrate moisture has been shown to influence fitness in various insect groups. High substrate moisture prolongs post-hatching survival in cicadas, with a greater proportion of juveniles reaching the adult phase [5]. Water availability in the oviposition substrate may also affect body size (e.g., as in house crickets [20]), and is likely correlated with survival probability [58]. Body size and mass in crickets are positively correlated with fecundity and desiccation resistance [23, 51, 59]. Further, female crickets prefer larger males [60, 61], probably because they are better competitors and tend to occupy territories with plentiful resources and favorable environmental conditions, such as grasshoppers [62].

For stridulating species, high moisture content may also facilitate higher fitness in males, as it allows males to produce louder calls. Moisture has been shown to directly affect stridulation rate in mole crickets [63]: wet soil is less porous and absorbs less sound, thus songs are louder and reach farther distances; these calls tend to attract more females [29]. In addition to enhancement of song and other fitness characteristics in males [64–67], females may also interpret higher male song intensity as a signal of availability of moist oviposition substrate [29]. Females can also detect substrate moisture through hygroreceptors on the ovipositor, antennae, and general body surface [68]. Thus, females use various mechanisms to evaluate substrate moisture, and can choose to oviposit eggs in the most suitable locations.

Low moisture level may also induce diapause, which is the interruption of embryogenesis due to unfavorable environmental periods [69, 70]. In katydids, moisture level is one of the factors that induces (during unfavorable conditions) and ends (when conditions become favorable) the physiological process of diapause [71]. Diapause is an important evolutionary strategy that allows populations to persist in partially unfavorable environments [31], increasing species geographic distribution [72].

Despite the preference for moist substrates, females also chose to oviposit on dryer substrates. Ovipositing in less moist substrates was higher in the field than in the lab, which can be due to the following mechanisms: (i) in the field, females could compare the experimental substrate with the surrounding forest soil, so that, whenever the surrounding soil was less moist than the experimental substrate, the female preferred the experimental substrate, or (ii) for a female to choose the moistest substrate in the field, it had to walk (or jump) further away (between 10 and 80 m), leading some females to oviposit before reaching the moistest substrates.

A further explanation would be that ovipositing in less moist substrates could potentially represent bet-hedging behavior, in which sites with unpredictable or variable environmental conditions favor genotypes that spread the risk of reproductive failure by utilizing a wider range of environmental conditions [73, 74]. Organisms lacking specific reproductive strategies have higher fitness in this case, due to an increase in the probability of offspring development in a changing environment [75]. Species that exhibit bet-hedging behavior have decreased fitness in the short-term, however, over a longer time period fitness is increased because populations have a lower probability of extinction due to environmental variability [76]. Female crickets that oviposit in both moist and dry substrates may thus have higher reproductive success in environments with highly variable moisture compared to females that restrict oviposition to substrates with similar moisture levels [77]. This may explain the presence of eggs in nearly all moisture levels in our experiments. An alternative explanation is that oviposition site selection may be influenced by a multifactorial decision making. For example, *Gryllus texensis* females trade-off preferences for oviposition substrate temperature with predation risk [11]. In our experiment, this trade-off may represent an exchange of suitable moisture for sites with lower perceived predation risk. This is because although moist substrates are highly suitable for cricket reproduction, they may also attract competitors and predators, leading some females to choose less suitable (i.e., dryer) sites for oviposition.

Crickets oviposit at greater soil depths in dry substrates to prevent desiccation [77], representing an additional bet-hedging mechanism. When substrate moisture is high, females tend to lay eggs in the surface layers because the risk of desiccation is low and development is faster due to high water absorption by eggs during embryogenesis (up to 100% of the egg’s weight) [41, 78, 79]. Additionally, laying eggs in shallower depths when the soil substrate has high moisture, could indicate a preference for intermediate moisture levels, avoiding excessive moisture in lower substrate layers, and eventual drowning of the eggs. Further, eggs laid at lower depths experience lower mortality, as newly hatched crickets can easily dig out of shallow substrate. Juveniles that hatch in dryer and deeper soils have more difficulty digging out of the soil, and survival rates are consequently reduced [5].

Although our results showed that litter crickets oviposit into substrates with a wide range of moistures, there was a clear preference for moister substrates, as evidenced by the linear relationship between oviposition and substrate moisture. Moisture inside the forest is variable; while certain areas can maintain moisture independent of precipitation [80] either by water retention or evaporation delay [81, 82], moisture levels at other sites may depend on rainfall. In the field experiment, females preferentially oviposited in containers with higher moisture levels, suggesting active search for substrates with higher moisture. The negative effect of evaporation on the number of oviposited eggs shows that along the oviposition period in the field experiment (48 h), females re-evaluated the substrate moisture each time they oviposited, because evaporation is a cumulative process that increases over exposure time. This result is counterintuitive considering the absence of effects of evaporation on oviposition probability. To explain this, we suggest that there may be two behavioral decision making phases. The first phase involves choosing the sites in which to oviposit, leading to the linear response of oviposition probability to moisture level (e.g., as seen in the field experiment). The second phase involves females revisiting previous oviposition sites for evaluation and, if substrate conditions are suitable, deposit additional eggs. The substrates with reduced moisture level due to evaporation would then be rejected, leading to fewer overall numbers of eggs. This may explain the observed negative effect of evaporation on numbers of oviposited eggs, but not on the probability of oviposition.

In the lab, female *Endecous chape* Souza-Dias & de Mello, 2017, *Eidmanacris meridionalis* Desutter-Grandcolas, 1995 and *Laranda meridionalis* Desutter-Grandcolas, 1994 were found to oviposit immediately after mating, then either mate again or feed, after which they return to the same substrate container for further oviposition (M. Fianco & N. Szinwelski, in preparation). For these species, the same substrate container was used by various females in subsequent oviposition events. The low number of oviposited eggs in our field and lab experiments is not consistent with the reported average number of eggs laid by litter crickets in the natural environment, which is estimated to vary from 60 to 1000 eggs per night [41]. Our study may thus underestimate the oviposition potential of litter crickets in the study sites. However, despite the lower numbers of eggs oviposited compared to that reported in other studies, we nonetheless found a strong preference for substrates with higher moisture.

There are several factors that may explain the low number of eggs in our study. First, cotton is not a common substrate for these insects, and would likely be ignored in the presence of other common oviposition substrates such as soil, twigs, and leaves [9, 41]. It is thought that female crickets can detect substrate texture prior to oviposition by use of sensory receptors in the palps and ovipositor [68]. When female do not find a suitable substrate, they may delay oviposition or reabsorb the eggs to avoid unnecessary energy expenditure [83] or predation [4], thereby maximizing their own survival. However, cotton is widely used in lab rearing of crickets and in scientific experiments [8, 11, 84] and, given the absence of alternative substrates, our study showed a clear pattern of female cricket preference for higher moisture substrates, a result in agreement with the common consensus that forest litter crickets are hydrophilic [85]. Parasitism might be another mechanism leading to low number of eggs. Parasitized female crickets have a reduced lifespan [86] and consequently lower egg laying frequency, in addition to lower egg numbers due to nutritional depletion or endocrine manipulation [87]. Parasitism was likely not a factor for females used in the lab experiment, as they were visually examined and determined to be healthy and injury-free, and during this time we detected no external parasites (acari or fungi). Further, females from the lab experiment were stored in ethanol solution after conclusion of the experimental period. Females infected with endoparasitic nematodes would likely have been detectable, as we expect these parasites to exit the host body immediately after immersion into ethanol (F. Farias-Martins, pers. obs. [88]), where they would have been detected. However, we do not know the endoparasite infection status of our field females, as they were not dissected. Finally, the low numbers of eggs in our study may be partially attributed to seasonality. In another tropical region of New Zealand, Blank and collaborators [89] showed that female *Teleogryllus commodus* lay a greater number of eggs (60–1000 eggs per day) from March to April, which is the favorable season due to having higher temperatures. In the following months, the authors observed a strong decline in oviposition (resulting in 0–9 eggs per day). Thus, there is strong seasonality to oviposition behavior in this species and region. Our crickets were collected in May, shortly before the beginning of the colder season (June to August are the coldest months in our study area [90]). Thus, the low number of eggs found in our experiment may reflect a decline in overall oviposition effort in association with the end of the more favorable season.

## Conclusion

Our study showed that ovipositing forest litter crickets prefer higher moisture substrates. Although the experiment was performed with crickets, we expect that high moisture substrate availability is a limiting factor for oviposition of several forest insect groups, particularly litter arthropods. Overall reduction in the availability of moist substrate, as is expected with global climate change, may reduce the abundance and geographical distribution of these organisms, thereby threatening populations due to decline in recruitment. Our results also indicate substrate moisture as an important dimension of the cricket ecological niche, and suggest that these organisms are particularly vulnerable to changes in climate leading to habitat drying.

## Supporting information

S1 Table File containing the raw data used in the analyzes. (XLSX)
